# Time course of Graves’ orbitopathy after total thyroidectomy and radioiodine therapy for thyroid cancer

**DOI:** 10.1097/MD.0000000000005474

**Published:** 2016-12-02

**Authors:** Camille Louvet, Annamaria De Bellis, Bruno Pereira, Claire Bournaud, Antony Kelly, Salwan Maqdasy, Beatrice Roche, Francoise Desbiez, Francoise Borson-Chazot, Igor Tauveron, Marie Batisse-Lignier

**Affiliations:** a CHU Clermont-Ferrand, Endocrinology Department, Clermont-Ferrand, France; b Department of Clinical and Experimental Medicine and Surgery “F. Magrassi, A. Lanzara,” Second University of Naples, Naples, Italy; c CHU Clermont-Ferrand, Biostatistics Unit (Clinical Research and Innovation Direction), Clermont-Ferrand; d Department of nuclear medicine, Hospices civils de Lyon, Groupement hospitalier Est, Bron, France; e Nuclear Medicine, Jean Perrin Cancer Center, Clermont-Ferrand; f UMR CNRS 6293, INSERM U1103, Génétique Reproduction et Développement, Université Clermont-Auvergne, Aubiere; g Department of Endocrinology, Hospices Civils de Lyon, Bron, Université Lyon I; h Lyon 1 University, CRCL, INSERM U1052, Lyon, France.

**Keywords:** Graves’ orbitopathy, radioiodine therapy, thyroid cancer

## Abstract

The risk of cancer is relatively higher in Graves’ patients presenting simultaneously with thyroid nodules. Radioiodine (RAI) therapy recommended in high-risk differentiated thyroid carcinoma may be associated with worsening of a pre-existing Graves’ orbitopathy (GO) or developing a new onset. The impact of RAI therapy in patients with differentiated thyroid cancer on the course of a pre-exisiting GO has not been specifically investigated.

The aim of this study is to assess the influence of RAI treatment administered for differentiated thyroid cancer on the course of a pre-existing GO.

This is a retrospective multicenter study including 35 patients from the University Hospital of Clermont-Ferrand (7 patients) and Lyon-Est (6 patients) in France and from a literature review published as case reports or studies (22 patients).

Seven patients exhibited a worsened pre-existing GO after total thyroidectomy followed by RAI treatment for thyroid cancer. Older men, those who initially presented with a lower clinical score of GO before RAI therapy, received higher doses of ^131^I especially when prepared with recombinant thyroid-stimulating hormone, and/or not prepared with glucocorticoids during RAI are at a higher risk to worsen their GO.

This study is the first and complete study collection. We describe worsening of GO in 20% of patients after RAI treatment for thyroid cancer and determine a pool of predictive factors.

## 1 Introduction

Graves’ orbitopathy (GO) affects 10% to 25% of patients with Graves’ disease (GD) and is influenced by nonpreventable factors, such as age, sex, and genetic predisposition, and exogenous factors such as cigarette smoking, poorly controlled thyroid dysfunction (both hyperthyroidism and hypothyroidism), and radioiodine (RAI) treatment.^[1,2]^ GO is an inflammation of extraocular muscles and peri-orbital connective tissue mediated by auto-antibodies against common antigens to both thyroid and orbit such as thyroid-stimulating hormone receptor.^[3,4]^

Considering the relationship between autoimmune ocular and thyroid disease, it has been suggested that eye disease should be improved by total thyroidectomy followed by RAI therapy.^[5–8]^ The rationale is based on removing both thyroid-orbit cross-reacting auto-antigens and autoreactive T lymphocytes following thyroidectomy. Conversely, several reports describe exacerbation of eye disease after iodine therapy suggesting that GO may worsen by the release of antigens and also by inducing hypothyroidism.^[9–11]^

The risk of cancer is relatively higher in Graves’ patients in the presence of an accompanying nodular disease.^[12,13]^ Moreover, some authors reported that thyroid cancer associated with GD seemed to be more aggressive.^[14,15]^ RAI therapy is recommended in high-risk differentiated thyroid carcinoma,^[16]^ but it might worsen or induce a new onset of GO.^[17]^ The impact of RAI therapy specifically in patients with differentiated thyroid cancer on the course of GO has not been well investigated.

Herein, we analyze the risk and the predictive factors of GO deterioration in a cohort of patients with a pre-existing GO who received RAI therapy for differentiated thyroid cancer.

## 2 Methods

### 2.1 Study design

This is a retrospective multicenter study that included patients with mild or moderate GO treated by thyroïdectomy and RAI for thyroid carcinoma between June 2001 and January 2014 from 2 different centers: University Hospital Center of Clermont-Ferrand and University Hospital Center of Lyon Est. We extended our cohort thanks to literature review to include the previously published case reports, cohorts, or trials. A National Library of Medicine MEDLINE search was performed using the following MESH terms (“Graves’ orbitopathy” or “Graves’ orbitopathy”) and (“radioiodine”) and (“ thyroid cancer” or “thyroid carcinoma”) for articles published during the period 1997 to 2015 and undertaken in July 2015. Only English and French language publications were considered. Only articles (including case reports, series of case reports, literature review, meta-analysis, letters to the editor) with a diagnosis of orbitopathy due to a GD and thyroid cancer treated by RAI therapy were retained.

All included manuscripts were reviewed blindly and successively read by 2 endocrinologists in order to complete the dataset. Only articles describing in details GO evolution in patients treated by RAI therapy for differentiated thyroid cancer were retained. Cases with no GO before RAI treatment and cases with GD treated with surgery and RAI without differentiated cancer were excluded. In each retained article, clinical, biological, and ophthalmological data were available and reportable.

We contacted the authors of the published articles, where appropriate, for further pertinent data.

This study is retrospective and a review of previously published date, so informed consent is not required.

### 2.2 GO evaluation

The ocular parameters were evaluated according to the European Group of Graves’ orbitopathy (EUGOGO).^[18]^ The severity and activity of the disease were assessed using the Clinical Activity Score (CAS). CAS is a model that assigns 1 point for each of the following items: spontaneous retrobulbar pain, pain on eye movement, eyelid erythema, eyelid edema, chemosis, conjunctival congestion, and swelling of the caruncle. A total score ≥3 was defined as active orbitopathy. We assessed the CAS before and after RAI for each patient.

Severity measures were in accordance with EUGOGO recommendations and included measurement of lid aperture; measurements of CAS, measurement of proptosis by Hertel exophthalmometer; evaluation of diplopia with subjective diplopia score; evaluation of corneal involvement; and evaluation of optic nerve involvement.

GO improvement was defined as changes in 2 or more of the following outcome measures in at least 1 eye, without deterioration in the opposite eye: reduction of CAS ≥2 points; reduction of eyelid width ≥2 mm; reduction in proptosis ≥2 mm; improvement in diplopia (disappearance/change in degree); and improvement in best corrected visual acuity (BCVA) by ≥2 lines on Snellen chart.

Conversely, GO worsening was defined as changes in 2 or more of the following outcome measures in at least 1 eye: increase of CAS ≥2 points; increase of eyelid width ≥2 mm; increase in proptosis ≥2 mm; worsening in diplopia (appearance/change in degree); and reduction in BCVA by ≥2 lines on Snellen chart. GO was considered stable in the absence of changes or when changes were smaller than any of the above-defined parameters.

### 2.3 Data assessment

The following data were assessed: age, sex, gender, smoking status, duration of GD before surgery, biological data as levels of FT4 (pmol/L), FT3 (pmol/L), thyroid-stimulating hormone (TSH) (UI/mL), TSH receptor antibodies (TRAK) before and after RAI (biological parameters were converted to fold normal values to standardize result), thyroid volume, indication of thyroidectomy (poor control of hyperthyroidism, intolerance to treatment, multi nodular goiter, volume goiter), management of GO before RAI (local treatment, surgery, radiotherapy, oral and/or intravenous glucocorticoid administration), cumulative dose of administered RAI (GBq), use of recombinant human TSH (RhTSH) stimulation or after thyroid hormone withdrawal (THW) before RAI, and thyroglobulin levels during follow-up, delay before worsening, and duration of follow-up.

### 2.4 Statistical analysis

All the analyses were performed using Stata software (version 13; StataCorp, College Station, TX) and were done for a 2-sided type I error of a = 5%. Baseline characteristics were presented as the mean ± standard deviation (SD), median (interquartile range) for continuous data (assumption of normality assessed by using the Shapiro–Wilk test), or as the number of patients and associated percentages for categorical parameters. Quantitative variables were compared between independent groups (deterioration or improvement/stabilization of GO) by Student *t* test or Mann–Whitney test if conditions of *t* test were not respected (normality and homoscedasticity analyzed using Fisher–Snedecor test). Comparisons between independent groups were done by Chi-square or when appropriate by Fischer exact test for categorical variables. The study of relationship between quantitative outcomes was performed using correlation coefficients (Pearson or Spearman according to statistical distribution). To take into account between and within-study variability due to the design of this study (meta-analysis on individual data), these analyses were completed using generalized linear mixed models (logistic for dichotomous dependent variable: evolution worsening or improvement/stabilization) to study the fixed effects, considering study as a random effect. Given the sample size, no meta-regression has been proposed in multivariate context. These analyses were completed by multidimensional factorial analysis (factorial mixed data analysis as FMDA to analyze assets as elements of qualitative and quantitative variables) to uncover the underlying relationships and structure (latent constructs) of a relatively large set between measured variable and to aggregate subjects into clusters such that each cluster represents a topic (deterioration or improvement/stabilization). In other words, FMDA was used to determine which parameters discriminate between natural evolutions (discrimination rules are based on linear combinations of the observed variables, called discriminant factors). The parameters selected to be included in the FMDA process were chosen according to univariate results and to clinical relevance. The analytical study of the equations involved shows that the discriminant factors minimize the Mahalanobis distance into each group and maximize the distance between groups, providing compact groups that are spread as much as possible in the space. The usual methodology is to transform the quantitative variables on categorical parameters categorizing them into classes to submit, ultimately, these new variables and the variables in an exploratory multiple correspondence analysis (MCA). This methodology is relatively easy to implement and can be used when sample size was sufficient (n > 100). Otherwise, the MCA could give unstable and nonrobust results. Also, it is interesting to retain, through methods such as FMDA, as is the quantitative variables, particularly in 2 situations: when the number of categorical variables is very small compared with the quantitative variables and when the sample size is low. The literature is relatively heterogeneous concerning the justification of sample size estimation in the context of this study. Finally, a sensitivity analysis has been proposed to measure the impact of missing data.

## 3 Results

**Figure 1:**
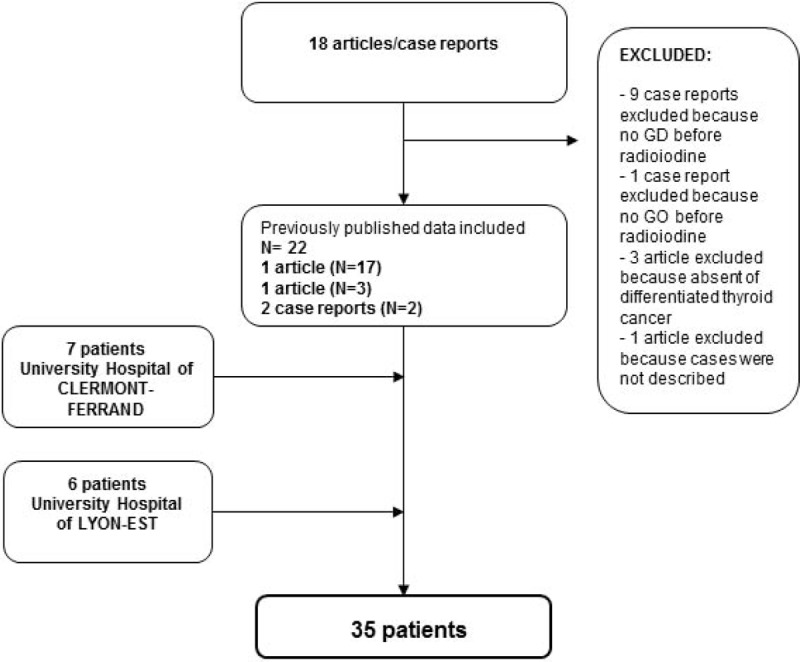
Flowchart of searched articles and case reports.

Seven patients with GO, treated both by thyroidectomy and RAI for incidental thyroid carcinoma, were included from the University Hospital of Clermont-Ferrand. Six cases came from the University Hospital of Lyon-Est. The number of cases with previously published data that met the inclusion criteria for this study was 22. They are from a case report series with 3 patients,^[19]^ a clinical trial with 17 cases published by De Bellis et al,^[20]^ and from 2 case reports.^[21,22]^ Finally, 35 patients were included for analysis (Fig. 1).

### 3.1 Characteristics of patients

The main characteristics of the included patients are summarized in Table 1.

**Table 1:**
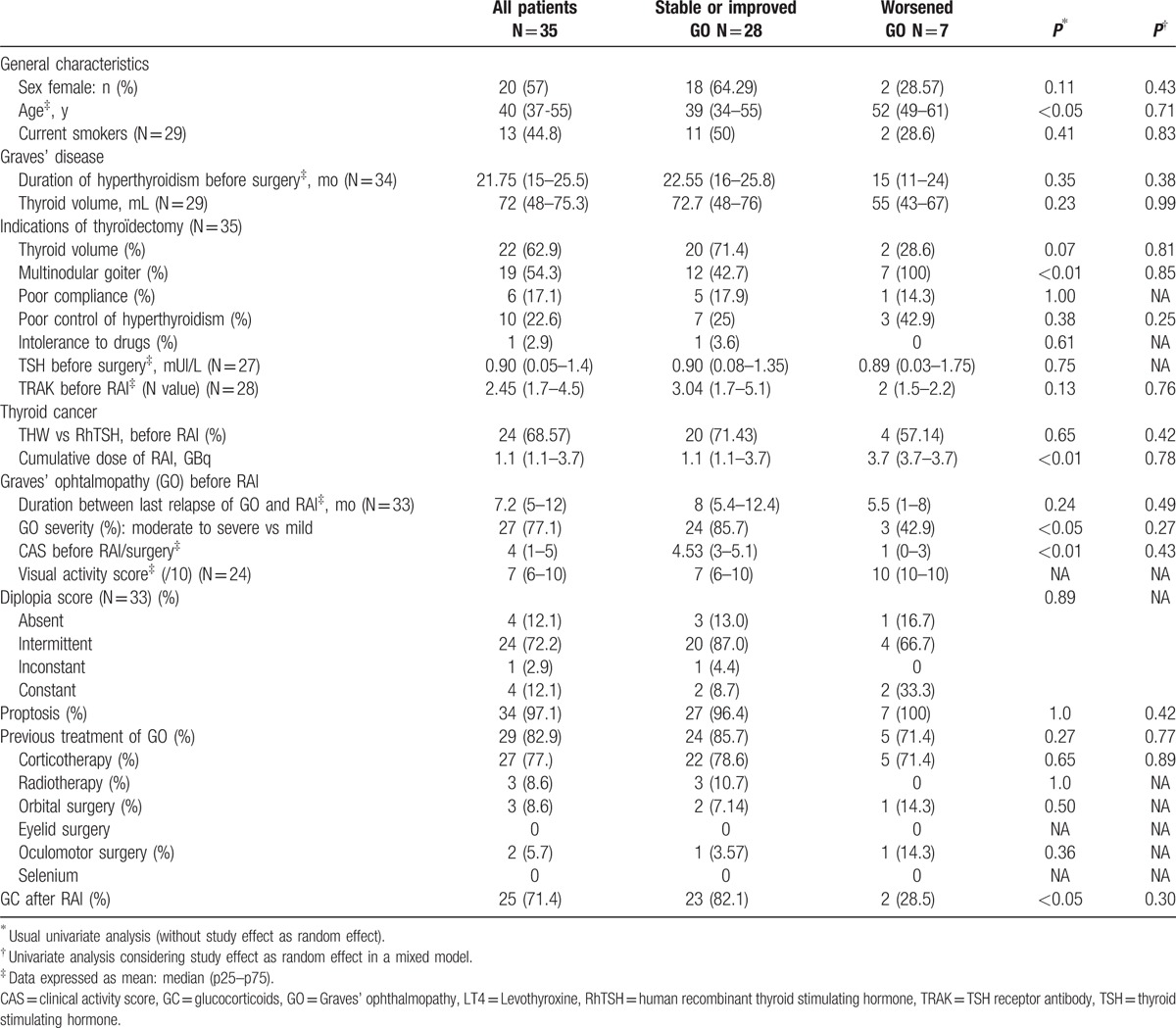
Characteristics of the study group.

This study included 20 women and 15 men. Mean age of patients was 44 ± 13 years. Thirteen (44.8%) patients were current smokers.

Multinodular goiter with median thyroid volume of 72 mL was the main indication of thyroidectomy. The median cumulative dose of RAI (indicated for differentiated thyroid cancer) was 1.1 GBq.

RAI was administered after RhTSH treatment in 11 patients (31.4%) and after THW in 24 (68.6%) patients.

Median TRAK levels before RAI was 2.45-fold the normal values. Median TSH levels before surgery were 0.90 mUI/L.

### 3.2 Ophtalmological data before RAI treatment

Median CAS of GO before RAI was 4 of 7 and 27 (77.1%) patients had moderate to severe GO before RAI.

Twenty-nine (82.8%) patients received specific treatment of GO before thyroidectomy with corticosteroid administration in 27 patients, radiotherapy in 3, orbital surgery in 3, and oculomotor surgery in 2 patients.

Twenty-five (71.4%) patients received prophylactic glucocorticoids after RAI treatment.

### 3.3 Outcomes

After RAI treatment, GO was improved in 19 patients (54.3%) and stabilized in 2 patients (5.7%), whereas it was worsened in 7 (20%) patients. Ophthalmologic data are insufficient for 7 patients to differentiate for “stable or improvement.”

The characteristics of patients with worsened GO are summarized in Table 2.

**Table 2:**
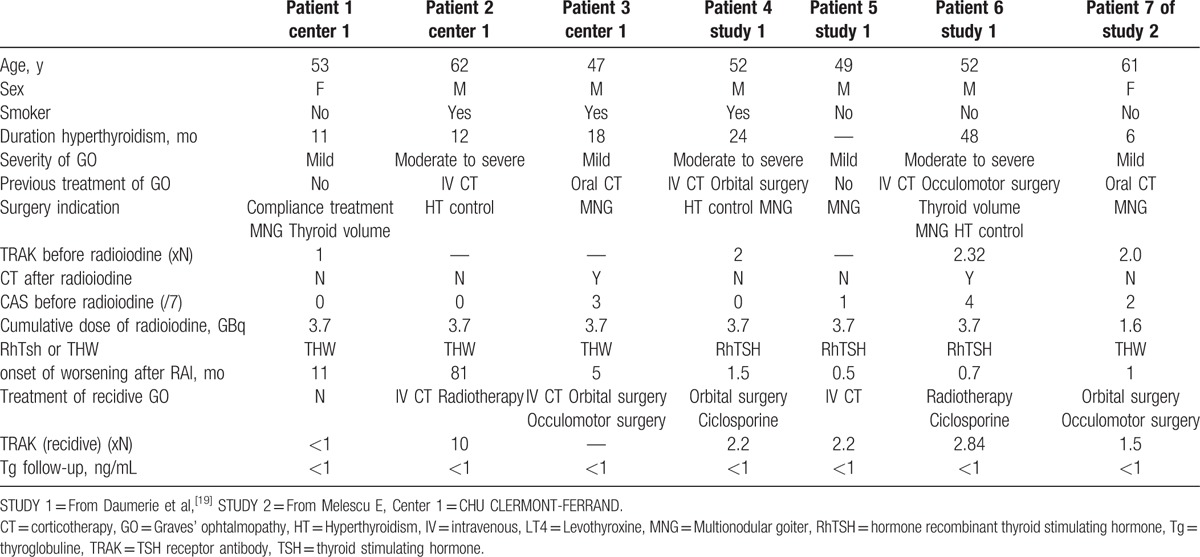
Characteristics of patients with GO worsening.

Median time onset of worsening was 1.5 months after RAI. Median duration of follow-up of patients was 6 years. Reactivation or aggravation of GO after RAI needed a complementary treatment of GO in 6 of 7 patients.

In usual univariate analysis, patients with worsened GO were statistically older (52 vs 39 years, *P* < 0.05) and were all operated because of a multi-nodular goiter (100% vs 42.9%, *P* < 0.01).

Mild GO according to EUGOGO classification (*P* < 0.05), less active according to CAS (median CAS = 1 vs 4.525, *P* < 0.01), and higher cumulative dose of RAI (3.7 vs 1.1 GBq, *P* < 0.01) were associated with an increased risk of worsened GO. Prophylactic corticosteroids after RAI were associated with a better ophthalmological prognosis (28.6% vs 82.1%, *P* < 0.05).

Patients with moderate to severe GO were more treated by prophylactic glucocorticoids after RAI (84% vs 50%, *P* = 0.054) and patients with active GO (CAS ≥3/7) were more treated by prophylactic glucocorticoids after RAI than patients with inactive GO (95.45% vs 36.36%, *P* < 0.001).

In univariate analysis considering study effect as random in a mixed model, there was no statistical difference between the 2 groups of patients (Table 1).

### 3.4 Factorial discriminant analysis

A predictive model for the principal variables was elaborated using a score derived by the factorial discriminant analysis (FDA) to determine which parameters discriminate between natural evolutions (discrimination rules are based on linear combinations of the observed variables, called discriminant factors). The parameters selected to be included in the FDA process were chosen according to univariate results and to clinical relevance.

**Figure 2:**
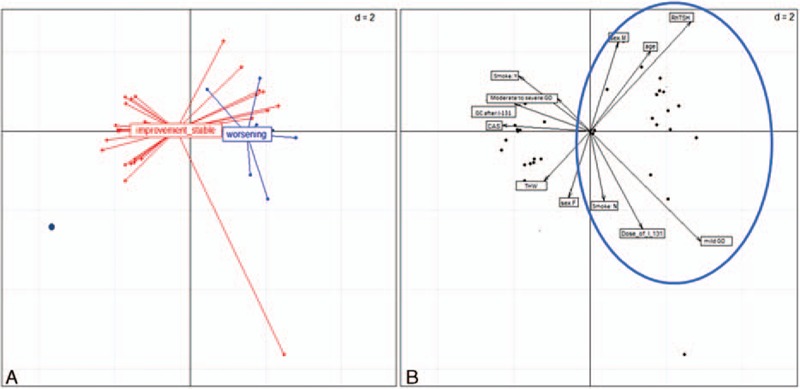
Results of factorial discriminant analysis of mixed data. (A) Patients representation showing 2 clusters: 1 with worsening GO after TTA (blue) another with improvement or worsening after TTA (red). (B) Variables representations showing one cluster with worsening GO (blue).

Figure 2A is the representation on a plane projection of discriminant variables (circle of correlations) and the Fig. 2B is the FDA patients’ map. The FDA defined 2 clusters of patients. The cluster of patients with deteriorated GO after RAI tended to be older men, with mild GO, prepared by RhTSH for RAI treatment and received a higher cumulative dose of RAI. The cluster of patients with improvement or stabilization of GO after RAI tended to be women with initial moderate to severe GO and higher CAS level who have received prophylactic glucocorticoids after RAI administration.

## 4 Discussion

Discovering thyroid cancer on histological examination of resected thyroid tissue of patient with GO is a difficult scenario for the clinicians. Indeed, when the risk of recurrence of thyroid cancer is high, total thyroid ablation (TTA) with RAI should be proposed to the patient. But the impact of RAI therapy in these patients on the course of a pre-existing GO had not been well investigated. The present multicenter retrospective study is, to our knowledge, the largest case series to report the evolution of GO after TTA for thyroid cancer. This work may help clinicians to identify patients at risk of worsening GO after RAI.

In the present study, among the 35 patients with preexisting GO, 7 patients worsened, 2 stabilized, and 19 patients improved their GO. The ophthalmological data were insufficient for 7 patients to estimate their evolution. One of the aims of our work was to determine the predictive factors of GO worsening after RAI. We identified that older men, patients treated with higher cumulative doses of RAI, patients prepared for RAI with RhTSH, and patients with mild or nonactive (CAS <3) GO are factors associated with GO worsening.

The present risk of worsening GO suffers from inherent biases that are usually associated with retrospective studies. We remain cautious and consider that the risk of aggravation is overestimated in our study, because the published case reports were mostly patients with worsened GO. Indeed, the clinicians have to consider this risk before iodine administration.

Interestingly, the majority of patients improved with TTA. These results are consistent with the hypothesis that reduction of thyroid tissue by TTA removes both autoreactive T lymphocytes and thyroid antigens. Few other studies have recently explored the topic of TTA to treat GO with RAI therapy in the absence of differentiated thyroid cancer.^[6–8]^ They suggested that GO outcome was more favorable in patients with TTA than in patients with thyroidectomy alone. Leo et al^[8]^ concluded that patients treated with TTA achieved a greater improvement in a shorter period of time than thyroidectomy alone. Notably, all patients included in these studies received 1.1 GBq of RAI and were treated by intravenous glucocorticoids after TTA.

In the context of thyroid cancer, the issues are more complex because the needed RAI doses are usually higher and repeated. Indeed, some case reports of worsening GO after TTA for thyroid cancer have been described in literature.^[19,21,22]^ Moreover, some cases of de novo GO occurrence after TTA for thyroid cancer in patients without GD before RAI are also published.^[23–31]^ Thus, the risk of appearance, aggravation, or improvement of GO in patients with thyroid cancer is unpredicted.

In usual univariate analysis, patients with worsening GO after TTA were more frequently older men. This is in agreement with previously published data. Indeed, in a retrospective study of 113 patients, Vannuchi et al^[32]^ showed that the reactivation of GO after RAI for GD was more prevalent in males and in older patients.

GO worsening was more frequent when higher cumulative doses of RAI were administered. To our knowledge, this topic has never been well investigated. Indeed, in the precedent prospective and retrospective studies, the dose of I 131 was usually fixed to 1.1 GBq. Meanwhile, patients treated by RAI for thyroid cancer are more likely to receive higher dose of RAI because of persistent thyroid tissue than patients without thyroid cancer. In our analysis, patient with deteriorated GO systematically received a cumulative dose superior to 1.1 GBq (median cumulative dose was 3.7 GBq). Furthermore, published cases reports with occurrence of de novo GO after TTA for thyroid cancer used always more than 1.1 GBq. For example, Woeber and Schwartz^[29]^ described a case of occurrence of de novo GO 5 months after the administration of a total cumulative dose of 12.95 GBq of RAI for metastatic thyroid cancer. So, it is suggested that the cumulative dose of RAI could influence evolution of GO after TTA.

Surprisingly, patients with worsened GO after RAI had less severe and less active GO before RAI than patients who remained stable or improved. This result may be explained by the fact that patients with moderate to severe and active GO (CAS ≥3/7) were more frequently treated by prophylactic glucocorticoids after RAI (moderate to severe vs mild: 84% vs 50%; *P* = 0.054, active vs inactive: 95.45% vs 36.36%; *P* < 0.001). In agreement with previous published studies,^[11,33–35]^ we confirm that prophylactic glucocorticoids after RAI prevent aggravation of GO. Furthermore, these results suggest that glucocorticoids should be systematically introduced to prevent the risk of GO worsening including patients with previous mild GO. Shiber et al^[35]^ suggested to administer a standard dose of prednisone (0.4–0.5 mg/kg body weight, tapered down and withdrawn after 3 months) to patients with mild to moderate GO, while a lower dose (0.2–0.3 mg/kg body weight, tapered down and withdrawn after 6 weeks) might be used in patients with even milder forms or in those who have no GO before RAI treatment but presenting risk factors for GO development.

Even if the results were not significant in the univariate analysis considering the study effect, results of FDA are consistent with the global univariate analysis. The cluster of patients with deteriorated GO after RAI tended to be older men, with mild GO, prepared by RhTSH for RAI treatment, and received higher cumulative dose of RAI.

Paradoxically, RhTSH before RAI is associated with GO aggravation. Indeed, hypothyroidism is associated with aggravation of GO^[36]^ and using RhTSH should prevent the risk of any detrimental ocular effects resulting from THW and consequent hypothyroidism. Moleti et al^[6]^ demonstrated that RhTSH in GO patients is safe and unaccompanied by significant adverse ocular effects. Our results should be interpreted cautiously; 3 of 7 patients with aggravation prepared for RAI by RhTSH are derived from the same study by Daumerie et al^[19]^ suggesting a *bias* linked to study effects in our analysis.

High TSH-receptor antibody titer is known to be an independent risk factor for GO outcome,^[37]^ but no studies found a correlation with TSH receptor antibody levels and progression of ophthalmopathy after RAI. In this study and in agreement with the previously published studies, TRAK levels before radiodine or surgery were not correlated to the risk of GO worsening.^[32,38,39]^ In our study, change of TRAK levels after RAI in patients with worsened GO were variables. Data concerning change in TRAK levels were available in only 4 patients of the 7 patients with worsened GO. In 2 patients (1 and 7), TRAK levels decrease after radioidine, whereas in 2 other patients (4 and 6), TRAK levels increase after TTA. The decrease of TRAK value may be explained by the fact that RAI is administered after total thyroidectomy. When RAI treatment is used for GD, an excessive release of thyroid antigens leading to an increased production of autoantibodies, such as TSH-R, occur.^[1]^ This, in turn, might induce eye injury. In our cohort, RAI is used to ablate remnant microscopic thyroid tissue. The TTA, by removing both thyroid orbit cross-reacting autoantigens and autoreactive T lymphocytes, is followed by decreased TRAK levels. Worsening of GO after TTA is thus not always a reflection of a worsening of auto-immunity. Maybe other unknown factors seem to be involved in the worsening of GO after TTA for thyroid cancer. The number of patient is not sufficient to conclude on the role of variation of TRAK level in this context.

In conclusion, our study describes cases of worsening of GO after TTA for thyroid cancer and determine a pool of predictive factors in favor of worsening. Deterioration seems independent of aggravation of auto-immunity. Special consideration for glucocorticoid treatment should be given especially in patients who will receive high doses of RAI, even in those with mild GO. Further prospective studies are needed to confirm our results.
